# The serine protease HtrA plays a key role in heat-induced dispersal of pneumococcal biofilms

**DOI:** 10.1038/s41598-020-80233-0

**Published:** 2020-12-31

**Authors:** Yashuan Chao, Caroline Bergenfelz, Renhua Sun, Xiao Han, Adnane Achour, Anders P. Hakansson

**Affiliations:** 1 Division of Experimental Infection Medicine, Department of Translational Medicine, Lund University, Malmö, Sweden; 2 Division of Infection Medicine, Department of Clinical Sciences, Lund University, Lund, Sweden; 3 Science for Life Laboratory, Department of Medicine, Karolinska Institute, Solna, Sweden; 4 Division of Infectious Diseases, Karolinska University Hospital, Solna, Stockholm Sweden

**Keywords:** Bacteriology, Biofilms, Infection

## Abstract

*Streptococcus pneumoniae* (the pneumococcus) colonizes the human nasopharynx by forming multicellular biofilms. Due to the high level of asymptomatic carriage, transition to infections, such as otitis media, pneumonia, sepsis, and meningitis, occurs often enough that the pneumococcus remains a major cause of disease and death globally. Virus infection and virus-induced responses, such as increased temperature (fever), trigger release of virulent bacteria from colonizing biofilms. The exact mechanisms involved in pneumococcal egress during biofilm dispersal remain unknown, although we hypothesize that disruption of the biofilm matrix encasing the bacteria is necessary. Here, we utilized established in vitro biofilm dispersal models to investigate the involvement of proteases in bacterial egress from pneumococcal biofilms. We demonstrate the importance of protease activity, both through increased bacterial release following addition of proteases and reduced heat-induced biofilm dispersal in the presence of protease inhibitors. We identify a key role for the surface-exposed serine protease HtrA, but not PrtA, in heat-induced biofilm dispersal. Bacterial release from *htrA-*negative biofilms was significantly reduced compared to wild-type isogenic strains but was restored and increased above wild-type levels following addition of recombinant HtrA. Understanding the specific mechanisms involved in bacterial egress may provide novel targets for future strategies aimed to specifically interfere with disease progression without disturbing nasopharyngeal biofilm colonization.

## Introduction

*Streptococcus pneumoniae* (the pneumococcus) is a frequent colonizer of the mucosal surfaces in the human nasopharynx. Colonization starts already within the first few weeks to months of life and persists in healthy individuals^1^. The prevalence of pneumococcal colonization is highest among children under the age of five and decreases into adulthood^2,3^, with asymptomatic colonization of the nasopharynx being the most common outcome after acquisition. However, in the presence of disease triggers, pneumococci are able to disseminate and survive in otherwise non-infected sites, such as the middle ears, lungs, and bloodstream, where pneumococcal disease manifests as acute otitis media, pneumonia, sepsis, and meningitis. Although infection rates are relatively low, the abundant colonization burden ensures that the pneumococcus remains a leading cause of these diseases globally and a major cause of death, especially in children and elderly; the most susceptible populations^4^.

Like many other respiratory pathogens, pneumococci form biofilms within the upper respiratory tract in humans, which can be recapitulated in various experimental model systems^5–9^. The propensity of biofilm bacteria for asymptomatic colonization is supported by several studies suggesting that biofilm-grown pneumococci exhibit attenuated virulence compared with broth-grown planktonic bacteria^9–11^. Colonization by pneumococci is thought to be a prerequisite for subsequent pneumococcal infection^12^. We have previously shown that exposure of pneumococcal biofilms to factors associated with viral infections, such as febrile-range temperatures and damage-associated molecular patterns (DAMPs), provokes egress of pneumococci from biofilms^11^. Among these stimuli, exposure to heat (the febrile temperature of 38.5 °C) alone promoted the greatest extent of biofilm dispersal and caused a phenotypic change in dispersed bacteria that was representative for the responses seen by other stimuli^11,13^. This adheres well with in vivo evidence showing that increased temperatures in sub-Saharan countries directly correlate with a more frequent transition of stable colonization into pneumonia, sepsis, and meningitis^14^. While biofilm dispersal induced by disease triggers results in the release of pneumococci with a distinct phenotype that is more invasive and inflammatory than both biofilm bacteria and broth-grown planktonic bacteria^11^, the underlying mechanisms of pneumococcal dispersal remain unknown.

Biofilm bacteria are encased within an extracellular matrix composed of DNA, proteins, carbohydrates, and other structural molecules^15^. Bacterial lysis is important for pneumococcal biofilm formation as pneumococci lacking the major autolysin LytA form poor biofilms with no matrix^8^, indicating that the material from lysed pneumococcal cells makes an important contribution to the biofilm matrix and its associated protection from antimicrobial agents^16^. It seems likely that molecules involved in the degradation of biofilm matrix macromolecules are responsible for and potentiate the dispersal process. In support of this notion, disassembly of bacterial biofilms with a focus on preventing biofilm formation or reducing biomass of existing biofilms has been successfully achieved by adding exogenous agents, including proteases, that target structural components of the biofilm matrix^17–21^. Still, the exact identity of the enzymes or molecules that are responsible for promoting biofilm egress during disease progression remains unclear. In our previous global transcriptome studies, pneumococci dispersed from biofilms by various triggers, including febrile-range temperatures, displayed an increased expression of the two surface proteases PrtA and HtrA^13^. Interestingly, of the two proteases, HtrA displays an increased activity with increasing temperature^22^.

In this study, we focused on the role of specific proteases in pneumococcal temperature-induced biofilm dispersal. We demonstrate that exogenous addition of proteases increased bacterial release from pneumococcal biofilms. Moreover, the presence of protease inhibitors prevented release of pneumococci from biofilms after exposure to febrile temperature. Altogether, these results indicate an important role for protease activity in pneumococcal biofilm egress. Using our transcriptional information combined with our established biofilm dispersal model, we identified HtrA as a key factor involved in bacterial egress from biofilms, whereas no such role was found for PrtA. Thus, interfering with molecules such as HtrA may constitute promising future approaches to specifically prevent transition from biofilm colonization to disease.

## Results

**Figure 1:**
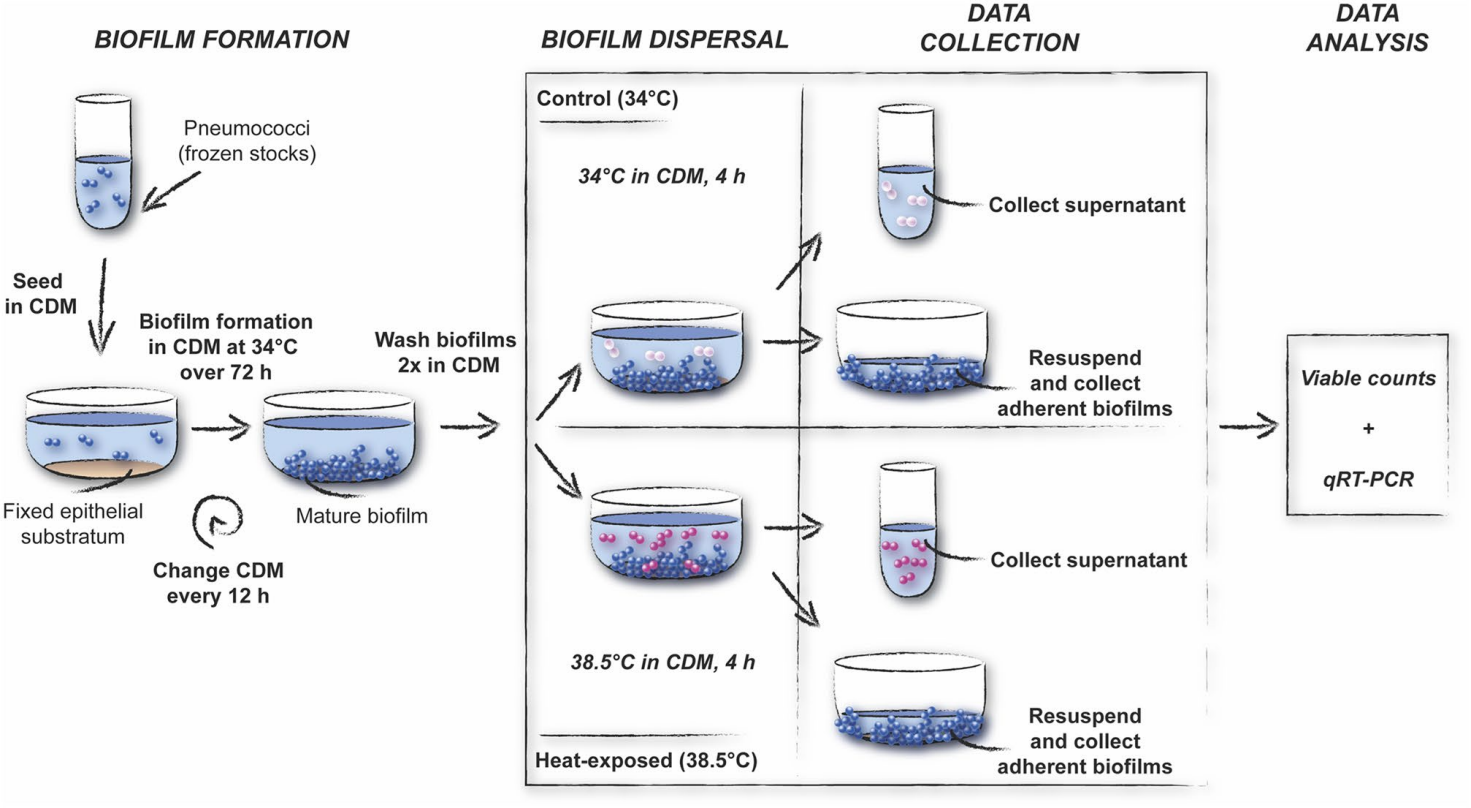
Schematic overview of the model system used in this study. Biofilms were formed in flat bottom wells over 72 h in CDM at 34 °C, with change of medium every 12 h. Biofilms were washed twice in CDM and then incubated in fresh medium at 34 °C (control; upper panel) or at 38.5 °C (lower panel) for 4 h. Supernatants containing released bacteria from control (34 °C) or heat-exposed (38.5 °C) biofilms were then collected and enumerated by viable plate counts. The remaining biofilms were resuspended and collected. Supernatant and biofilm fractions were saved for gene expression analysis. The example shown is for heat-induced biofilm dispersal. Similar methods were used for inhibitor experiments and exogenous protease experiments.

Based on pneumococcal biofilm disassembly studies^18,19,21^ and our previous findings demonstrating an upregulation of surface proteases associated with heat-induced dispersal^13^, we here focused our attention on investigating protease activity as a mechanism of bacterial release from biofilms by using our established in vitro biofilm and biofilm dispersal models (Fig. 1).

### Protease activity plays a key role in biofilm dispersal

**Figure 2:**
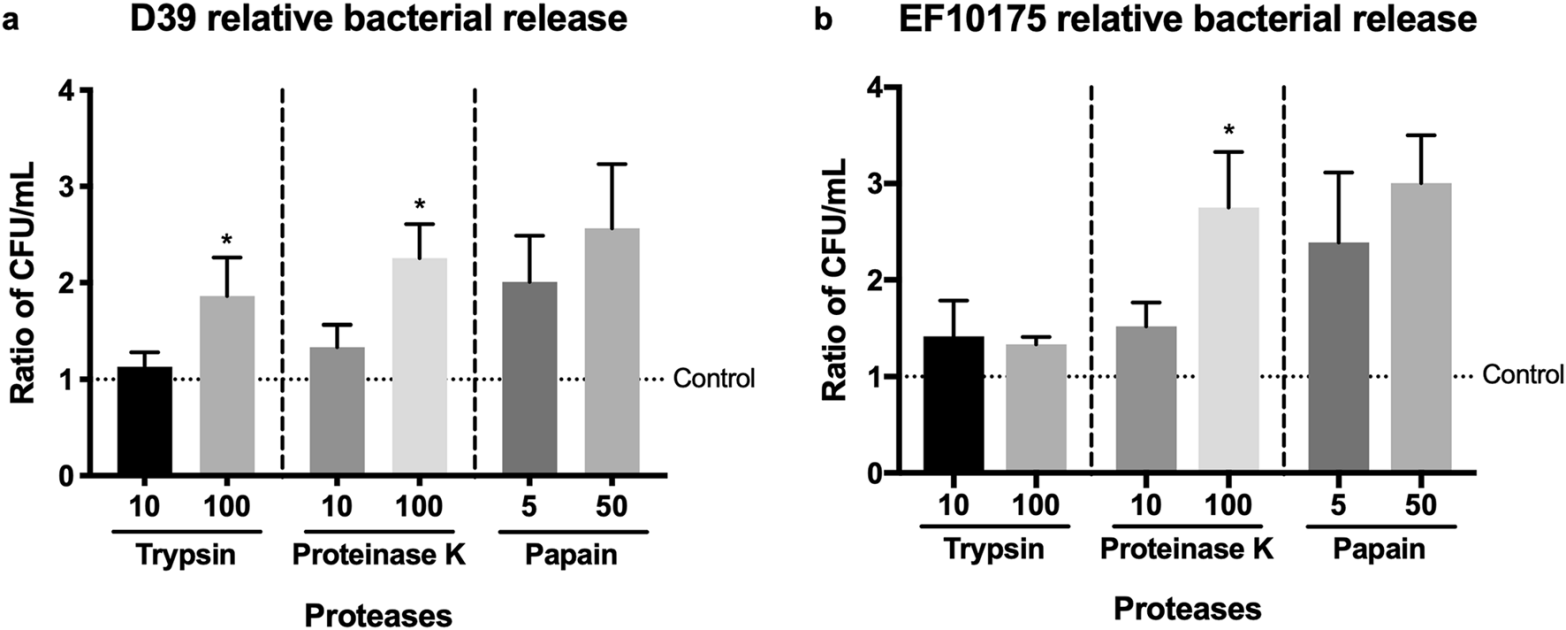
Role of protease activity in biofilm dispersal. (**a**) D39 and (**b**) EF10175 biofilms were exposed to indicated concentrations (μg/ml) of proteases at 34 °C for 2 h. Supernatants containing the released bacteria were enumerated by viable plate counts. The relative bacterial release is shown as a ratio of the CFU/ml of enzyme-exposed compared to control without proteases, i.e., normalized to control. For reference, the average bacterial release for the control was approximately 1.7 × 10^7^ CFU/ml for D39 and 3.7 × 10^8^ CFU/ml for EF10175. Data represent triplicates from four separate experiments, with the mean and SEM displayed. Statistical analysis was performed using Kruskal–Wallis test with Dunn’s multiple comparisons test; *P < 0.05.

To investigate whether exogenously added proteases alone were sufficient for bacterial egress, mature pneumococcal biofilms were washed and continuously exposed to the nasopharyngeal temperature of 34 °C with or without indicated proteases for 2 h (Fig. 2). The serine proteases trypsin and proteinase K as well as the cysteine protease papain increased pneumococcal release from biofilms formed by both the laboratory strain D39 and the clinical isolate EF10175 (Fig. 2), indicating that extracellular proteases can cause release of pneumococci from biofilms.

**Figure 3:**
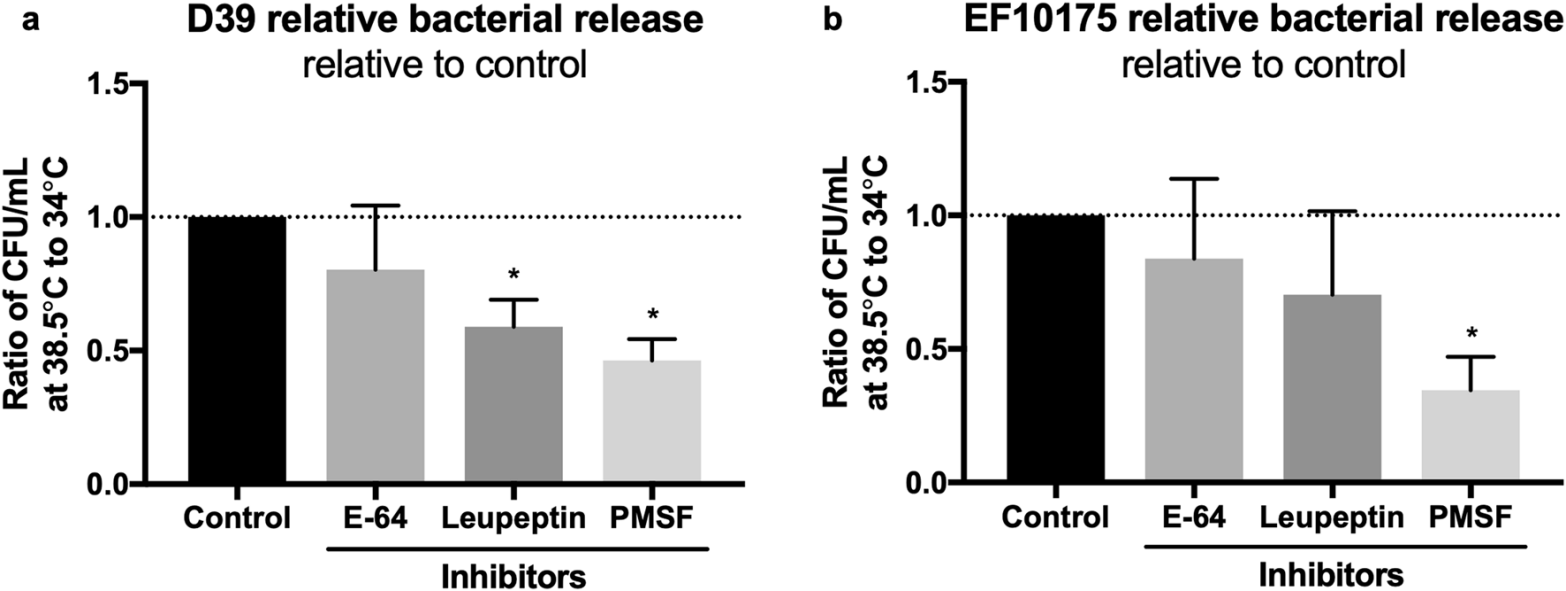
Role of protease activity in heat-induced biofilm dispersal. (**a**) D39 and (**b**) EF10175 biofilms were exposed to protease inhibitors PMSF (20 μM), leupeptin (10 μM), or E-64 (10 μM) at 34 °C or 38.5 °C for 4 h. Supernatants containing the released bacteria were enumerated by viable plate counts. The relative bacterial release is shown as a ratio of the CFU/ml at 38.5 °C compared to 34 °C and then normalized to the control without inhibitor. For reference, the average bacterial release for the control was approximately 6.5 × 10^7^ CFU/ml for D39 and 9.7 × 10^7^ CFU/ml for EF10175, which was 3.6 times or 2.8 times higher than the release at 34 °C, respectively. Data represent triplicates from four separate experiments, with the mean and SEM displayed. Statistical analysis was performed using Mann–Whitney U test by comparing to the control; *P < 0.05.

As exposure to the febrile temperature of 38.5 °C resulted in a phenotypic change representative of other disease triggers in dispersed bacteria^11,13^, we next investigated the role of protease activity in heat-induced dispersal. Biofilms were exposed to 38.5 °C for 4 h or continually exposed to 34 °C in the presence or absence of protease inhibitors and bacterial release into the supernatant was determined by viable plate counts (Fig. 3). In biofilms formed by strain D39, presence of either serine protease inhibitors PMSF or leupeptin significantly reduced heat-induced dispersal as compared to control biofilms, whereas presence of the cysteine protease inhibitor E-64 did not have any significant effect (Fig. 3a). Similar results were observed in biofilms formed by the strain EF10175, although a significant decrease in heat-induced dispersal was only seen in the presence of PMSF (Fig. 3b). Interestingly, passive bacterial release as part of normal turnover of bacterial biofilms at 34 °C was not inhibited by exposure to protease inhibitors (data not shown), suggesting a different mechanism of release. Together, our results demonstrate a role for proteases, primarily serine proteases, in heat-induced pneumococcal dispersal from biofilms but not in the release of bacteria during normal biofilm turnover.

### Pneumococcal mutants lacking HtrA and PrtA form biofilms comparable with the isogenic wild-type strain

We have previously demonstrated that 134 genes were differentially regulated in heat-dispersed pneumococci compared with biofilm counterparts of the clinical isolate EF3030^13^. Among the differentially expressed genes, those encoding the pneumococcal surface-exposed serine proteases HtrA and PrtA were upregulated in the heat-dispersed population^13^. In order to investigate whether these enzymes were involved in bacterial egress from biofilms upon exposure to heat, we tested strains lacking the expression of these enzymes in our biofilm dispersal model.

**Figure 4:**
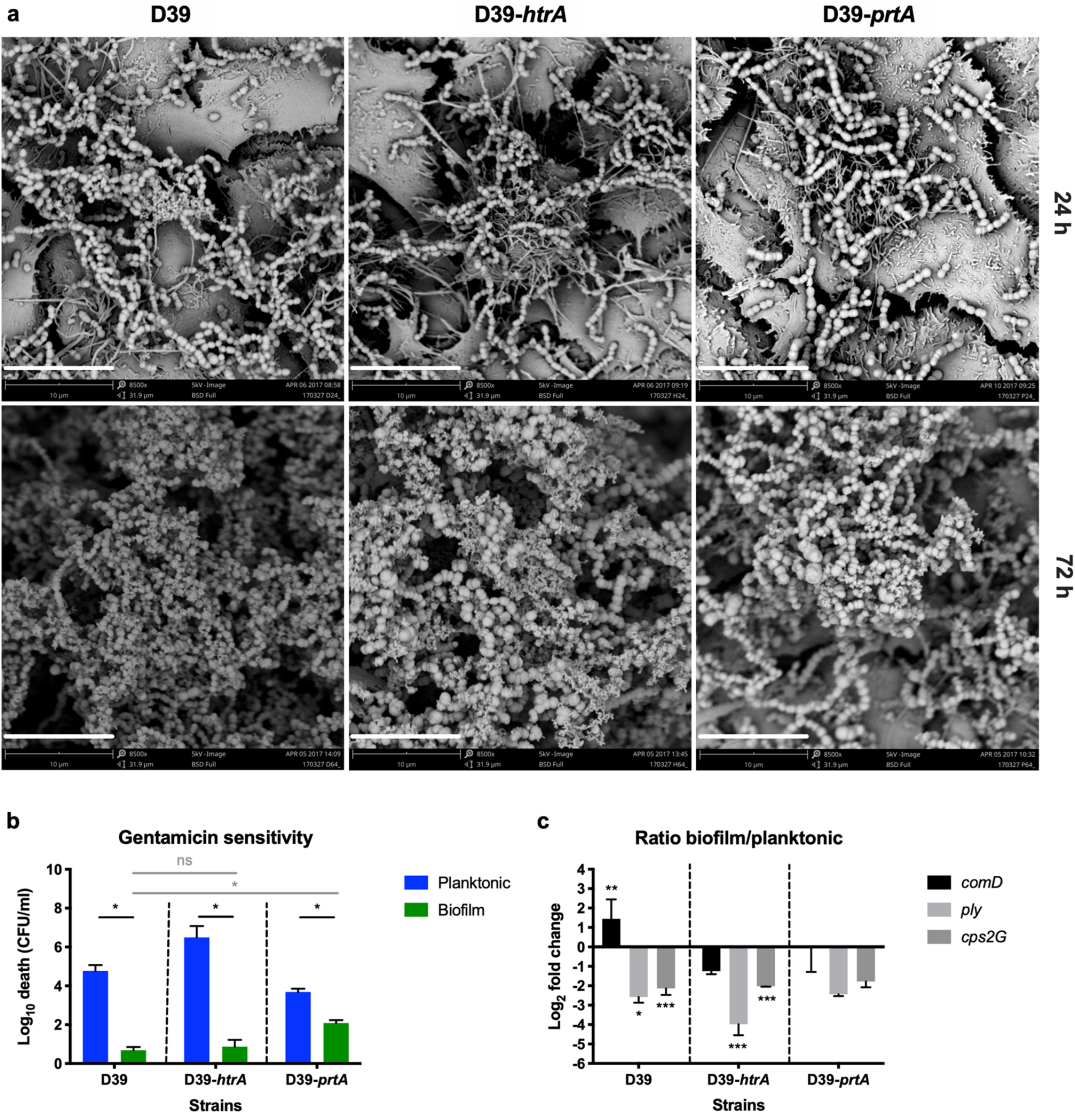
Biofilm formation by protease mutants. (**a**) Biofilm morphology was detected at 24 h (upper panels) and 72 h (lower panels) by scanning electron microscopy. The white scale bar represents 10 μm. (**b**) Wild-type and mutant D39 biofilm and planktonic bacteria were exposed to 500 μg/ml gentamicin for 3 h and the sensitivity to the antibiotic was determined by viable plate counts. (**c**) Relative gene expression of wild-type and mutant D39 as a ratio of biofilm bacteria to broth-grown planktonic bacteria. Data represent triplicates from four separate experiments, with the mean and SEM displayed. Statistical analysis was performed using Mann–Whitney U test (black) or Kruskal–Wallis test with Dunn’s multiple comparisons test (grey) (**b**) and two-way ANOVA with Dunnett’s multiple comparisons test (**c**); *ns* not significant, **P < 0.01, ***P < 0.001.

First, we validated that the mutated strains maintained a similar capacity to form biofilms as the wild-type strain (Fig. 4 and Fig. S1a). Analysis by scanning electron microscopy revealed that both wild-type D39 bacteria and *htrA*- and *prtA*-negative strains in the D39 background formed biofilms with similar structures and morphologies that developed equally well over 72 h (Fig. 4a). Antibiotic tolerance is a common characteristic of mature, functional biofilms. We therefore routinely assess pneumococcal biofilm formation by biomass and tolerance to the antibiotic gentamicin, where pneumococci in mature biofilms are less sensitive to gentamicin killing than broth-grown planktonic pneumococci^8^. Wild-type D39 and the protease-negative strains formed structurally robust biofilms of similar biomass (Fig. S1a) that were significantly less sensitive to gentamicin killing as compared to the respective planktonic bacteria (Fig. 4b). Unlike D39-*htrA* biofilms, the functionality of D39-*prtA* biofilms was less developed based on their significantly increased sensitivity to gentamicin compared to wild-type D39 (Fig. 4b). Reduced sensitivity to gentamicin killing of biofilm bacteria can result either from altered metabolic activity and/or from formation of a physical barrier (i.e., by the biofilm matrix) to the antibiotic. Here, we observed that bacterial death induced by gentamicin increased from less than 1 log_10_ to approximately 3.5 log_10_ when D39 biofilms were mechanically disrupted and resuspended prior to treatment with gentamicin (data not shown), suggesting that the physical barrier of intact biofilms contributes to protection against the antibiotic. Finally, the biofilm bacteria showed a similar regulation of known differentially expressed genes^11,13^ as compared to their planktonic counterparts, with downregulation of the classical virulence factors *ply* (pneumolysin) and *cps2G* (capsule) (Fig. 4c). These results indicate that mutations of *htrA* or *prtA* did not affect the capacity of the pneumococcal strain D39 to form biofilms.

### Pneumococcal mutants lacking HtrA display impaired ability to disperse in response to heat

**Figure 5:**
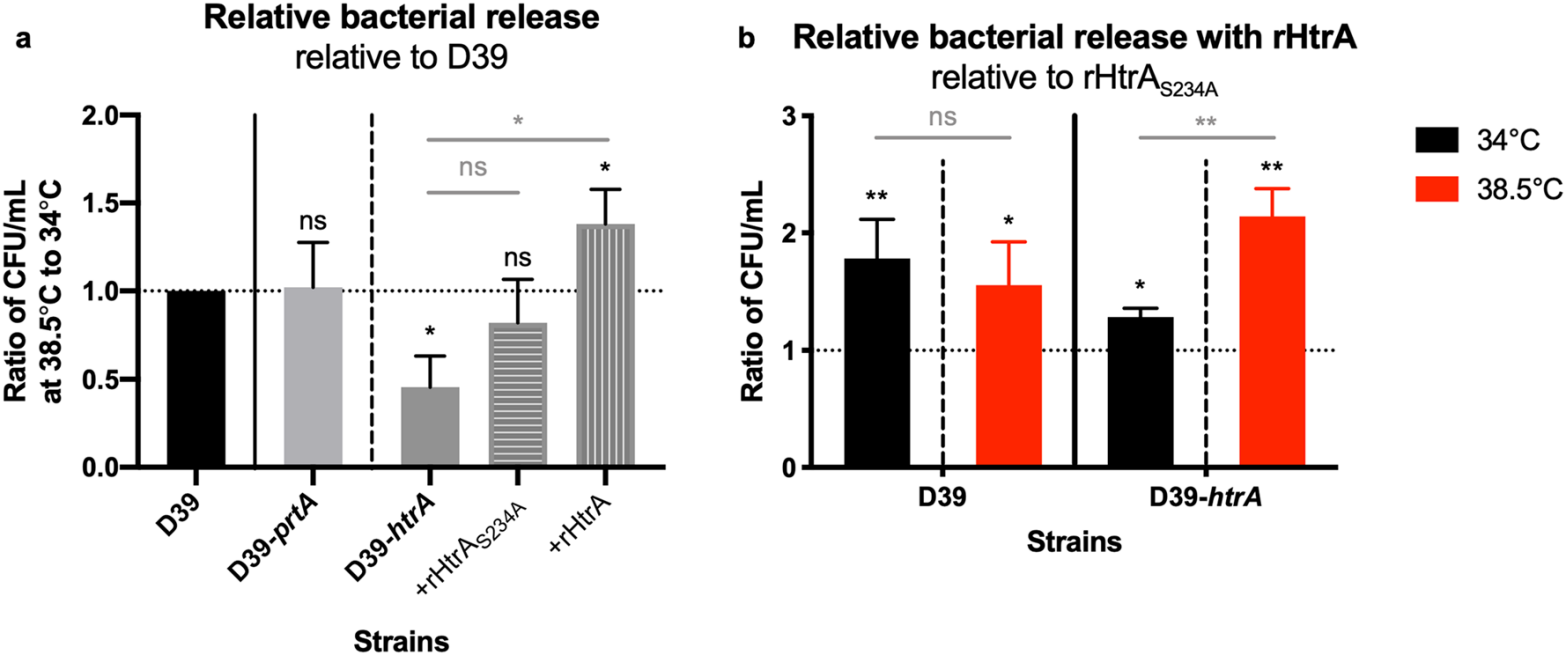
Impact of protease mutations and complementation with recombinant HtrA on heat-induced biofilm dispersion. Wild-type and mutant D39 biofilms were washed in CDM and exposed to 34 °C (control) or 38.5 °C (for heat-induced dispersal) for 4 h. Supernatants containing the released bacteria were enumerated by viable plate counts. (**a**) The relative bacterial release is shown as a ratio of the CFU/ml at 38.5 °C compared to 34 °C and then normalized to the wild-type strain. For reference, the relative bacterial release for wild-type D39 was approximately 1.1 × 10^8^ CFU/ml at 38.5 °C, which was 5.2 times higher than at 34 °C. Data represent duplicates or triplicates from three to four separate experiments, with the mean and SEM displayed. Statistical analysis was performed using Mann–Whitney U test by comparing to the wild-type strain (black) or Kruskal–Wallis test with Dunn’s multiple comparison test to the D39-*htrA* strain (grey). (**b**) The relative bacterial release is shown as a ratio of the CFU/ml with addition of rHtrA compared to with addition of rHtrA_S234A_ and then normalized to the latter. Data represent duplicates from three separate experiments, with the mean and SEM displayed. Statistical analysis was performed using Mann–Whitney U test by comparing to addition of rHtrA_S234A_ (black) or comparing between 34 and 38.5 °C within a strain (grey); *ns* not significant, *P < 0.05, **P < 0.01.

To test the role of HtrA and PrtA in heat-induced biofilm dispersal, biofilms of the wild-type D39 strain and its respective protease mutants were washed and exposed to the febrile temperature 38.5 °C for 4 h or continually exposed to 34 °C. The mutant strain lacking HtrA, but not the strain lacking PrtA, released significantly less bacteria in response to heat than wild-type D39 pneumococci (Fig. 5a). The role of HtrA in heat-induced biofilm release was confirmed using the *htrA*-negative mutant strain in the clinical isolate EF10175 background (Fig. S2). The *htrA*-negative strain displayed a reduction in heat-induced biofilm dispersal (Fig. S2d), while displaying comparable biofilm characteristics (Fig. S2a–c) with regards to biomass, decreased gentamicin sensitivity, and gene expression profiles as wild-type EF10175. No significant changes in expression were seen in the assayed genes between dispersed and biofilm bacteria for either the wild-type or mutant D39 bacteria (Fig. S1b), consistent with previous data for this strain. In EF10175 (serotype 19F), similar patterns of gene expression were seen between strains, with upregulation of *ply* (pneumolysin) and significant downregulation of *comD* (competence) in dispersed bacteria compared to biofilm bacteria (Fig. S2e), comparable to our previous experimental data with another 19F serotype clinical isolate EF3030^11,13^.

To assess the role of HtrA in the heat-induced release of pneumococci from biofilms, HtrA-negative biofilms were supplemented by addition of exogenous recombinant pneumococcal HtrA (rHtrA). The functional activity of purified wild-type rHtrA and rHtrA_S234A_ (harboring an inactivating point mutation in the active site) was verified by cleavage of β-casein (Fig. S3). Addition of rHtrA to *htrA*-negative D39 biofilms exposed to 38.5 °C resulted in a significantly increased release of bacteria compared with heat-exposed *htrA*-negative D39 biofilms alone (Fig. 5a). The observed release with rHtrA was also significantly higher compared with the heat-induced release of wild-type D39 (Fig. 5a). The HtrA-induced egress of *htrA*-negative D39 biofilm bacteria required protease activity as addition of rHtrA_S234A_ did not induce any significant release above either wild-type or *htrA*-negative D39 (Fig. 5a).

Bacterial release due to the addition of rHtrA was significantly increased not only at 38.5 °C but also at 34 °C in both the wild-type and the *htrA-*negative D39 strains (Fig. 5b). However, a significant increase of bacterial release at 38.5 °C compared to 34 °C was only observed with the *htrA*-negative strain and not with the wild-type strain (Fig. 5b). HtrA appears to have a specific role during heat-induced bacterial release, as passive bacterial release at 34 °C was similar between wild-type and *htrA*-negative D39 strains (Fig. S4). Interestingly, the highest bacterial release of about 1.3 × 10^8^ CFU/ml was reached when both HtrA (or rHtrA) and an elevated temperature of 38.5 °C were combined (Fig. S4). This further showed that addition of rHtrA did not contribute significantly to heat-induced release of wild-type D39 biofilm bacteria but was required for a similar level of heat-induced release of the HtrA-negative biofilm bacteria.

Altogether, our results suggest that protease activity plays a key role in heat-induced dispersal of pneumococci and demonstrate that the surface-exposed serine protease HtrA is a major contributor and key molecule during the release of pneumococcal biofilm bacteria in response to febrile-range temperature.

## Discussion

Asymptomatic pneumococcal colonization in the nasopharynx always precedes the development of potentially lethal diseases such as pneumonia, meningitis, and sepsis^12^. We have previously shown that pneumococci colonize the nasopharynx as complex bacterial communities or biofilms^8^. We have also shown that perturbation of the nasopharyngeal environment, including febrile-range temperature, causes pneumococcal egress from the biofilm community and dissemination of virulent organisms to other sites in the body where they can cause disease^11^. However, how pneumococci sense these changes and egress to enable spread from sessile biofilms to cause infection remains unknown.

In order to spread and potentially cause disease, pneumococci, as well as many other bacterial species, must make their way out of the matrix that embeds the bacteria in the colonizing biofilm. Biofilm dispersal and turnover is a general phenomenon for bacterial biofilms that have been studied in a number of bacterial species^23–27^. Similar to our observations in pneumococci^13^, dispersed *Pseudomonas aeruginosa* bacteria display a changed expression profile of genes different from both biofilm and planktonic bacteria^25^. However, with a few exceptions, the specific mechanism(s) of the natural dispersal process and its association with disease progression have not yet been well assessed. In *Haemophilus influenzae* biofilms, mutation of a nuclease decreases biofilm dispersal, suggesting an important role for DNA in biofilm integrity by this organism^26^. For *Streptococcus pyogenes*, the cysteine protease SpeB plays a role in dispersal of bacteria from biofilms and subsequent disease progression^28^. These studies propose a role for integral components of the biofilm matrix and degradative enzymes in the bacterial dispersal process.

The mechanisms involved in pneumococcal dispersal and egress during disease progression as well as biofilm turnover are still not well defined. However, in light of studies in other species, we hypothesized that enzymes that degrade the constituents of the matrix are likely involved in biofilm dispersal. It has been suggested that proteins may dominate over carbohydrates and DNA in some biofilms and that extracellular proteins may link bacteria to the surrounding matrix^15^. Therefore, as for *S. pyogenes*, cleavage of proteins could have a great potential for the release of bacteria from biofilms via degradation of the matrix.

Here, we demonstrate that addition of both exogenous serine and cysteine proteases induces release of bacteria from biofilms, suggesting that digestion of proteins alone in the extracellular matrix that encompasses the biofilm could release bacterial organisms. Additionally, and importantly, even though addition of exogenous proteases of both types caused bacterial biofilm release, only inhibition of serine protease activity, but not cysteine protease activity, significantly reduced release of pneumococci from biofilms in response to heat exposure. Although inhibition of serine protease activity significantly reduced heat-induced bacterial release from biofilms, the release was not abolished, suggesting that other processes likely also contribute to bacterial release. Infectious triggers, such as virus infection, DAMPs, and increased temperature, cause drastic changes in the gene expression of exposed biofilm populations^13^ and could alter bacterial activity and metabolism, their interactions with host tissues, as well as the expression of other factors involved in breakdown of the so far relatively uncharacterized extracellular matrix. This is, in our opinion, an interesting avenue for continued research and will be investigated in more detail in future studies.

Interestingly, protease inhibitors did not impair passive release during normal biofilm turnover at the nasopharyngeal temperature of 34 °C. This suggests that serine protease activity is associated primarily with changes in the colonizing environment, such as increased temperature, and is required for release of bacteria during transition to disease but is not responsible for normal bacterial turnover of colonizing biofilms. As exogenous proteases induced biofilm release at 34 °C but protease inhibition was ineffective, this also suggests that the intrinsic expression of proteases in biofilms at the nasopharyngeal temperature is most probably low.

We have previously identified several genes that were differentially regulated in heat-dispersed pneumococci compared with biofilm and broth-grown bacteria^13^. Among the genes that were upregulated in response to heat, two encoded the surface-exposed serine proteases HtrA and PrtA. Both of these factors play an important role during pneumococcal infection^29–31^ and especially HtrA, which is conserved among bacterial species, is also important for virulence in several other organisms^32–35^. However, the specific role of either protease in biofilm formation and dispersal has not been investigated.

In our present study, mutation of *htrA* or *prtA* did not affect the capacity of strains to form biofilms. However, the *htrA*-negative strains were significantly hampered in their ability to disperse in response to heat. This suggests that HtrA, but not PrtA, is involved in heat-induced pneumococcal dispersal from biofilms. The underlying mechanism is likely related to the protease function of HtrA that may be directly involved in recognition and cleavage of matrix components surrounding the pneumococci. This protease activity may also be increased at a higher temperature. HtrA contains a trypsin-like domain and a PDZ domain^29,36^, the latter of which mediates specific protein–protein interactions. It has been proposed that ligand binding at the PDZ domain may induce conformational changes that then transmit to the protease domain, thereby activating the proteolytic activity^36^. HtrA in *Escherichia coli* (DegP) and in other organisms functions as a chaperone involved in folding and processing of extracellular proteins during protein secretion, as well as a protease associated with degradation of misfolded proteins, the latter gaining significant importance at elevated temperatures^37,40^. The specific targets for HtrA are mostly unclear, although DegP from *E. coli* can degrade misfolded MalS, an alpha-amylase family protein^37^. Studies with pneumococcal HtrA have also indicated both chaperone and proteolytic activities with competence peptide as one potential proteolytic target^31,38–40^, as well as involvement in resistance to elevated temperature and oxidative stress^31^.

In an attempt to reconstitute the inhibited heat-induced biofilm release in *htrA*-negative biofilms, we produced and isolated recombinant pneumococcal HtrA (rHtrA) and the inactive version, rHtrA_S234A_, with a serine to alanine substitution in the active site. Using these two proteins, we were first able to complement HtrA-deficiency by addition of exogenous rHrtA. We observed significantly increased bacterial release of *htrA*-negative D39 biofilm bacteria after addition of rHtrA at an elevated temperature. This complementation required functional protease activity as the inactive rHtrA_S234A_ variant failed to complement HtrA-deficiency in the *htrA*-negative strain. Increased release was also detected for HtrA-negative bacteria at 34 °C, although this release was significantly lower than at 38.5 °C. These results demonstrate that addition of exogenous proteases can induce bacterial release from biofilms regardless of temperature and also indicate that the activity of HtrA may increase with increased temperature, consistent with HtrA being a heat-associated protease in several bacterial species. Interestingly, bacterial release from wild-type D39 biofilms was increased to the same degree at both 34 °C and 38.5 °C in the presence of rHtrA. This is most likely due to a “maximum” release already established at 38.5 °C for the wild-type strain expressing HtrA.

Since the serine protease encoded by *htrA* is surface-exposed on the pathogen, another intriguing avenue of future research is to evaluate its therapeutic and preventive potential. HtrA has already been used in preliminary vaccine studies and has been shown to protect against invasive pneumococcal disease in animals^41^. Whether HtrA immunization will specifically target the disease-causing (dispersed) pneumococcal population while allowing the asymptomatically colonizing pneumococci to maintain their niche in the nasopharynx, similar to our studies with other molecules upregulated in dispersed bacteria^41,42^, will be of interest to investigate. Similarly, interfering with HtrA activity has the potential to therapeutically inhibit transition from biofilm colonization to disease. In conclusion, we have demonstrated that protease activity is important for heat-induced release of pneumococci from biofilms and that the surface-exposed serine protease HtrA plays a key role during this process.

## Materials and methods

### Reagents

Cell culture media and supplements as well as trypsin were purchased from GE Healthcare Life Sciences. Reagents for chemically-defined medium for pneumococcal growth (CDM^16^), proteinase K (from *Tritirachium alba*), papain (from papaya latex), phenylmethanesulfonyl fluoride (PMSF; broad serine protease inhibitor), leupeptin (serine and cysteine protease inhibitor), E-64 (cysteine protease inhibitor), lysozyme, mutanolysin, β-casein, sodium dodecyl sulfate (SDS), TRI reagent, Isopropyl-β-d-thiogalactoside (IPTG), NaCl, and imidazole were purchased from Sigma-Aldrich. Gentamicin, HEPES, and DNase I were purchased from Thermo Fisher Scientific. Ni-NTA agarose was purchased from Qiagen (Hilden, Germany). Coomassie Brilliant Blue R-250 was purchased from Bio-Rad.

### Cells

The mucoepidermoid bronchial carcinoma cell line NCI-H292 (ATCC CCL-1848) was grown in RPMI-1640 with l-glutamine and supplemented with 10% fetal bovine serum, 100 U/ml penicillin, 100 μg/ml streptomycin, and 1% sodium pyruvate at 37 °C in the presence of 5% CO_2_. Cells were seeded into 24-well cell culture plates and grown until confluent. The monolayer was fixed with 4% paraformaldehyde (PFA), washed with PBS, and then stored in PBS at 4 °C until use.

### Bacterial strains

Strains used in this study are specified in Table 1. All pneumococcal strains were grown in CDM from frozen glycerol stocks. Viable plate counts were performed on blood agar. Pneumococci were verified by sensitivity to optochin after growth on blood agar plates in the presence of optochin discs^43^ (Sigma).

**Table 1:** Strains used in this study.

Strain or plasmid	Characteristic	References
***S. pneumoniae***		
D39	Wild-type, serotype 2	^44^
D39-*htrA*	D39, replacement of *htrA* with Janus cassette, Kan^R^	This study
D39-*prtA* (MJ04.2)	D39, insertional inactivation of *prtA*, Erm^R^	^30^
EF10175	Wild-type, serotype 19F	^45^
EF10175-*htrA*	EF10175, replacement of *htrA* with Janus cassette, Kan^R^	This study
Rx1-*htrA* (TH7774)	Rx1, replacement of *htrA* with Janus cassette, Kan^R^	^46^
***E. coli***		
TH8796	BL21, pET22b::*htrA*, Amp^R^	This study^a^
TH8930	BL21, pET22b::*htrA*_*S234A*_, Amp^R^	This study^a^

^a^Isolates were kindly provided by Dr. Jing-Ren Zhang, Tsinghua University, School of Medicine, Beijing, China.

### Construction of D39-*htrA* and EF10175-*htrA* mutants

A PCR product was generated from strain Rx1-*htrA* using primers HtrA-up and HtrA-down and transformed into strains D39 and EF10175 according to^47^. Transformants were selected by kanamycin resistance on blood agar plates containing 400 µg/ml of kanamycin and verified for insertion of the cassette at the correct site by PCR using primer Kan (within the kanamycin cassette) and primer HtrA-verif (outside of the fragment used for cloning). Besides being verified genetically, the strains were also verified through a lack of expression of the respective gene by qRT-PCR (data not shown). See Supplementary Table 1 for primer sequences.

### Biofilm formation and assessment

Biofilm formation was performed as previously described^16^. Briefly, frozen stocks of pneumococci were diluted in CDM to approximately 10^5^ CFU/ml, seeded onto PFA-fixed epithelial cells in 24-well plates, and grown at 34 °C, 5% CO_2_ over 72 h, replacing with fresh media every 12 h. A temperature of 34 °C simulates the measured nasopharyngeal temperature^48^. To assess biomass and antibiotic sensitivity, biofilms were exposed to 500 μg/ml gentamicin at 34 °C for 3 h, with PBS alone as a control for total initial biomass. As a comparison, planktonic bacteria grown at 37 °C in CDM to an optical density of 0.50–0.55 at 600 nm (approximately 2–3 × 10^8^ CFU/ml) were used. Biofilms were detached from the wells by scraping, sonicating three times (0.5 s each) in a water bath sonicator, and pipetting vigorously to ensure a homogenous solution. Biomass in CFU/ml was determined by viable counts on blood agar plates. To assess biofilm formation, the gentamicin sensitivity and gene expression in biofilm bacteria were compared with planktonic bacteria of respective strains.

### Biofilm dispersal models

Exposure to exogenous proteases: biofilms were washed twice with CDM and incubated in the presence of indicated proteases at 34 °C for 2 h. Exposure to heat: Biofilms were washed twice with CDM and incubated at 34 °C (control) or 38.5 °C for 4 h with or without indicated inhibitors or recombinant HtrA. The inhibitor concentrations were determined in an initial screen and concentrations were used where inhibition was detected without any effect on bacterial viability (data not shown). A vehicle control was used with PMSF, as this inhibitor was initially dissolved in ethanol. In both models, bacteria from both the supernatants (released bacteria) and the resuspended biofilms were analyzed by viable plate counts and stored at − 80 °C until later use for gene expression analysis.

### Scanning electron microscopy (SEM)

The SEM protocol used here has been previously described in detail^16^. In brief, biofilms were formed as described above except pneumococci were seeded onto round glass coverslips covered with a pre-fixed substratum of confluent NCI-H292 cells. Biofilms were fixed using 2.5% glutaraldehyde, 0.075% ruthenium red, and 0.075 M lysine acetate in 0.1 M sodium cacodylate buffer. Lysine and ruthenium red fixation has been shown to retain carbohydrate structures and improve preservation of biofilm structures^49^. Samples were washed in 0.075% ruthenium red in 0.2 M sodium cacodylate buffer and dehydrated with an ascending ethanol series from 50% (v/v) to absolute ethanol prior to critical point drying with carbon dioxide, with absolute ethanol as the intermediate solvent. Samples were then mounted onto aluminum holders, painted with silver around the sides (for better conductivity), sputter coated with 20 nm palladium/gold, and examined in a DELPHI correlative light and electron microscope (Phenom-World, IQ Biotechnology Platform, Infection Medicine, Lund University).

### RNA isolation and qRT-PCR

RNA was isolated from biofilm, supernatant (released bacteria), and planktonic samples. A detailed protocol for RNA isolation from pneumococcal cells is available^16^. Briefly, samples were subjected to enzymatic, chemical, and mechanical lysis. RNA was isolated using Zymo-Spin™ IIC columns (Zymo Research), subjected to DNase I digestion, then further purified via phenol–chloroform extraction, then stored at − 80 °C until use. RNA purity was verified by spectrophotometry as well as PCR for DNA contamination. RNA integrity was analyzed by running the RNA in a 1% agarose gel. cDNA was prepared by using the Bio-Rad iScript™ cDNA synthesis kit according to the manufacturer’s instructions. cDNA amplification was quantified by using the Applied Biosystems ViiA 7 Real-Time PCR System in the presence of SYBR green (Maxima SYBR Green/ROX qPCR Master Mix; Thermo Scientific). See Supplementary Table 1 for primer sequences. Relative gene expression values were calculated as described^50,51^, normalized to two stably expressed reference genes, *gyrA* and *16S*. The data shown here is the calculated log_2_ fold change between indicated bacterial populations.

### Production of recombinant HtrA

Plasmids containing the gene encoding pneumococcal full-length *htrA* (TH8796) or its active site mutant *htrA*_S234A_ (TH8930) cloned into the pET22b vector were kindly provided by Dr. Jing-Ren Zhang (Tsinghua University, School of Medicine, Beijing, China). The signal peptide of HtrA (or HtrA_S234A_), KHLKTFYKKWFQLLVVIVISFFSGALGSFS, was removed from the construct encoding full-length HtrA (or HtrA_S234A_). See Supplementary Table 1 for primer sequences. Cloning was performed following the FastCloning technique protocol^52^ and verified by sequencing. Poly-histidine-tagged (6xHis-) HtrA (or HtrA_S234A_) was expressed in *Escherichia coli* BL21-DE3 induced with 1 mM Isopropyl-β-d-thiogalactoside (IPTG) for 4 h at 37 °C and purified using Ni-NTA agarose with the buffer composed of 20 mM HEPES (pH 7.5), 150 mM NaCl, and imidazole according to the manufacturer’s instructions.

### HtrA protease activity

The activity of HtrA was determined using β-casein as substrate, essentially as described^22,53,54^. The proteolytically active (rHtrA) and inactive (rHtrA_S234A_) proteins (4 µM) were mixed with β-casein (22 µM) in 50 mM HEPES buffer (pH 6.5) containing 100 mM NaCl and incubated at 37 °C for 240 min. Samples were taken at 0, 30, 60, 90, 120 and 240 min, separated on 10% polyacrylamide gels, and stained with Coomassie Brilliant Blue R-250 (0.1% w/v in 10% glacial acetic acid, 50% methanol). Gel images were taken using a Gel Doc XR + system (Bio-Rad).

### Statistical analysis

Statistical analyses were performed using the GraphPad Prism 9 software. Comparisons were analyzed for statistical significance using Mann–Whitney U test, Kruskal–Wallis test with Dunn’s multiple comparisons test, or ordinary two-way ANOVA with Dunnett’s multiple comparisons test. Results were deemed significant for comparisons where *P* < 0.05.

## Supplementary Information

Supplementary Information.

## Data Availability

The datasets generated during and/or analysed during the current study are available from the corresponding author on reasonable request.
