# Supplementary material for: The serine protease HtrA plays a key role in heat-induced dispersal of pneumococcal biofilms
Source: Sci Rep. 2020 Dec 31;10:22455. doi: 10.1038/s41598-020-80233-0 (PMC7775458; doi:10.1038/s41598-020-80233-0)
Supplement: Supplementary file 1 — Supplementary Information. [file 41598_2020_80233_MOESM1_ESM.pdf]

SUPPLEMENTARY MATERIAL

The serine protease HtrA plays a key role in heat-induced dispersal of pneumococcal biofilms

Yashuan Chao, Caroline Bergenfelz, Renhua Sun, Xiao Han, Adnane Achour, and Anders P. Hakansson

SUPPLEMENTARY FIGURE LEGENDS AND SUPPLEMENTRY TABLES

Supplementary Figure 1. Impact of protease mutations on strain D39. Wild-type and mutant D39 (**a**) total initial biofilm biomass and (**b**) relative gene expression as a ratio of heat dispersed bacteria to biofilm bacteria. Data represent triplicates from four separate experiments, with the mean and SEM displayed. Statistical analysis was performed using Kruskal-Wallis test with Dunn's multiple comparisons test (**a**) and two-way ANOVA with Dunnett's multiple comparisons test (**b**); ns = not significant.

Supplementary Figure 2. Impact of protease mutations on strain EF10175. (**a**) Wild-type and mutant EF10175 total initial biofilm biomass. (**b**) Wild-type and mutant EF10175 biofilm and planktonic bacteria were exposed to 500 μg/ml gentamicin for 3 h and the sensitivity to the antibiotic was determined by viable plate counts. (**c**) Relative gene expression of wild-type and mutant EF10175 as a ratio of biofilm bacteria to broth-grown, planktonic bacteria. (**d**) Wild-type and mutant EF10175 biofilms were washed in CDM and exposed to 34°C (control) or 38.5°C (for heat-induced dispersal) for 4 h. Supernatants containing the released bacteria were enumerated by viable plate counts. The relative bacterial release is shown as a ratio of the CFU/ml at 38.5°C compared to 34°C and then normalized to the wild-type strain. For reference, the relative bacterial release for wild-type EF10175 was approximately 1 x 10^8 CFU/ml at 38.5°C that was approximately 2.3 times above the release at 34°C. (**e**) Wild-type and mutant EF10175 relative gene expression as a ratio of heat dispersed bacteria to biofilm bacteria. Data represent triplicates from four separate experiments, with the mean and SEM displayed. Statistical

analysis was performed using Mann-Whitney U test (**a**, **b**, and **d**) and two-way ANOVA with Dunnett's multiple comparisons test (**c** and **e**); ns = not significant, * = $P < 0.05$, *** $P < 0.001$.

Supplementary Figure 3. Degradation of β-casein by rHtrA. The proteolytically active (wild-type rHtrA; 4 μM) and inactive (S234A-mutant rHtrA, 4 μM) recombinant HtrA proteins were incubated with β-casein (22 μM) in 50 mM HEPES buffer (pH 6.5) containing 100 mM NaCl and incubated at 37°C for 240 min. Samples from various time points (0, 30, 60, 90, 120, 240 min) were separated on 10% polyacrylamide gels, and stained with Coomassie Brilliant Blue R-250 to detect degradation. The letter B in the legend denotes a lane where β-casein was separated without addition of rHtrA. The letter M denotes the marker with bands of 70, 55, 40, 35, 25, and 15 kDa (top to bottom).

Supplementary Figure 4. Bacterial release with or without elevated temperature and/or recombinant HtrA. Wild-type and mutant D39 biofilms were washed in CDM and exposed to 34°C (control) or 38.5°C (for heat-induced dispersal) for 4 h with exogenously added recombinant HtrA (rHtrA; 11 μM) or recombinant HtrA harboring a mutation at the active site ($HtrA_{S234A}$; 11 μM). Supernatants containing the released bacteria were enumerated by viable plate counts. Bacterial release is shown as CFU/ml. Data represent duplicates from three separate experiments, with the mean and SEM displayed. Statistical analysis was performed using Mann-Whitney U test (*black*) or Kruskal-Wallis test with Dunn's multiple comparisons test (*grey*); ns = not significant, * = $P < 0.05$.

SUPPLEMENTARY TABLES

Supplementary Table 1. Primers used in this study.

Primer	Sequence (5' → 3')	Reference
htrA-negative mutants		
HtrA-up	GAT CCC AGC AGG ATT CGA ACC	This study
HtrA-down	TTC TAA ATC ACC TGA AC	[1]
Kan (DAM345)	CAG GAG ACA TTC CTT CCG TAT CTT	[2]
HtrA-verif	CAA CAG CCT GAA ATA AGG CTG	This study
qRT-PCR		
comD F	GGT TCG TAT CAT GAG CGT TT	[3]
comD R	CCT GAA GGA GTC ATC GTC AT	[3]
cps2G F	CGT GAT GCA GAT GTA GTA ATT GCG	[3]
cps2G R	TGT AAG TGG CAA GCG ATA CGA TGC	[3]
ply F	CAG CTA CCC GAT GAG TTT GTT	[4]
ply R	TGC TCC AGG ATA GAG GCG ACT	[4]
Recombinant HtrA		
rHtrA 1	ATC ACC CAG TTG ACC CAG AAA AGT AGT GTA AAC AAC TCT AAC AAC	This study
rHtrA 2	CTG GGT CAA CTG GGT GAT CAT ATG TAT ATC TCC TTC TTA AAG TTA AAC	This study

Supplementary Figure 1

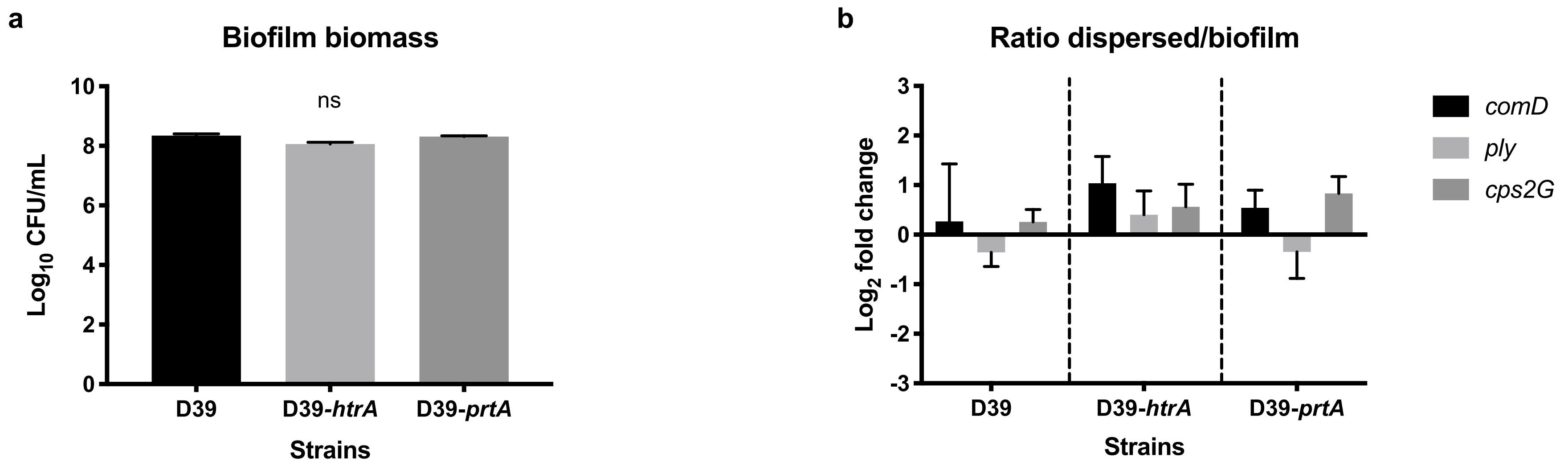

Supplementary Figure 2

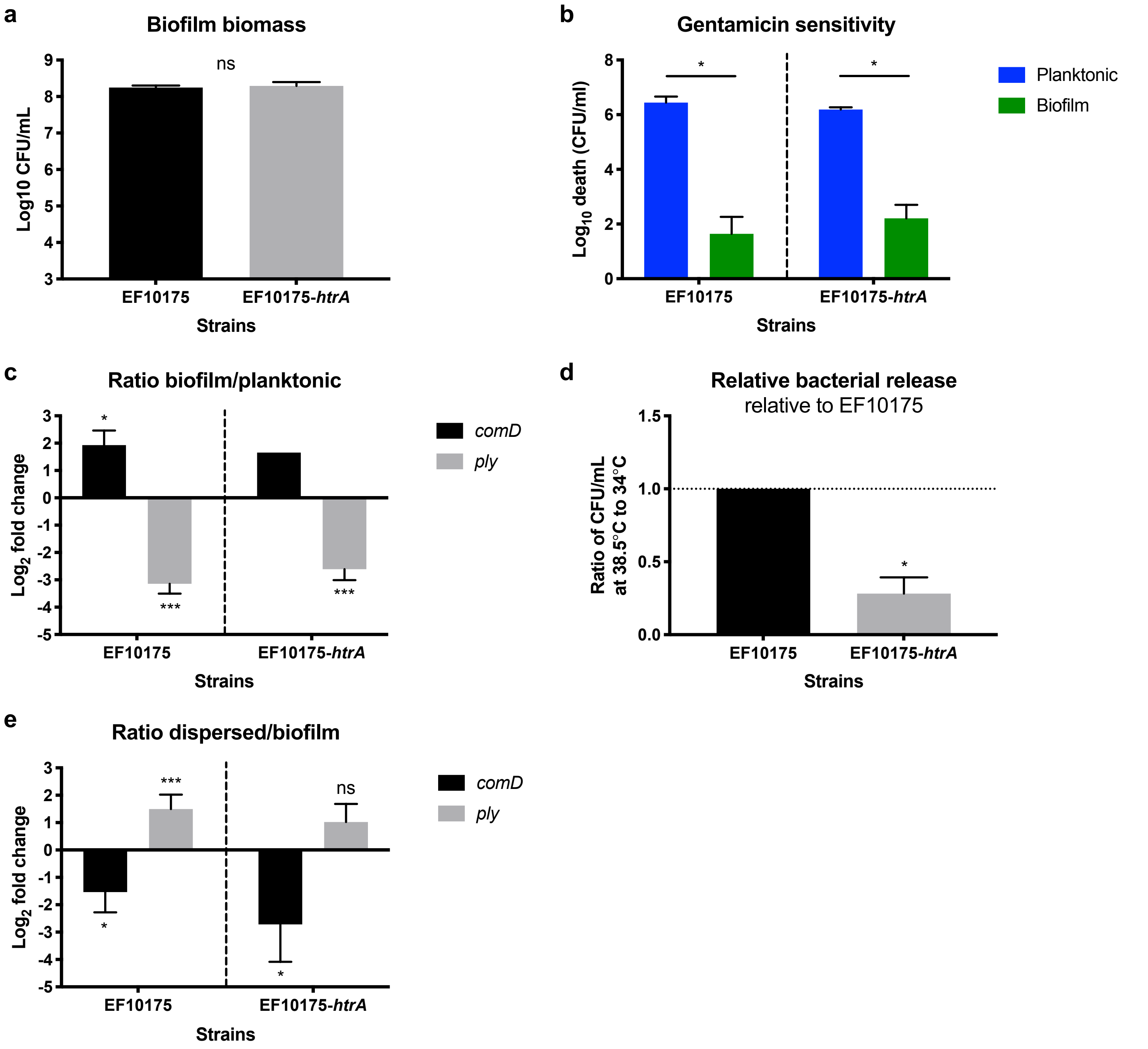

Supplementary Figure 3

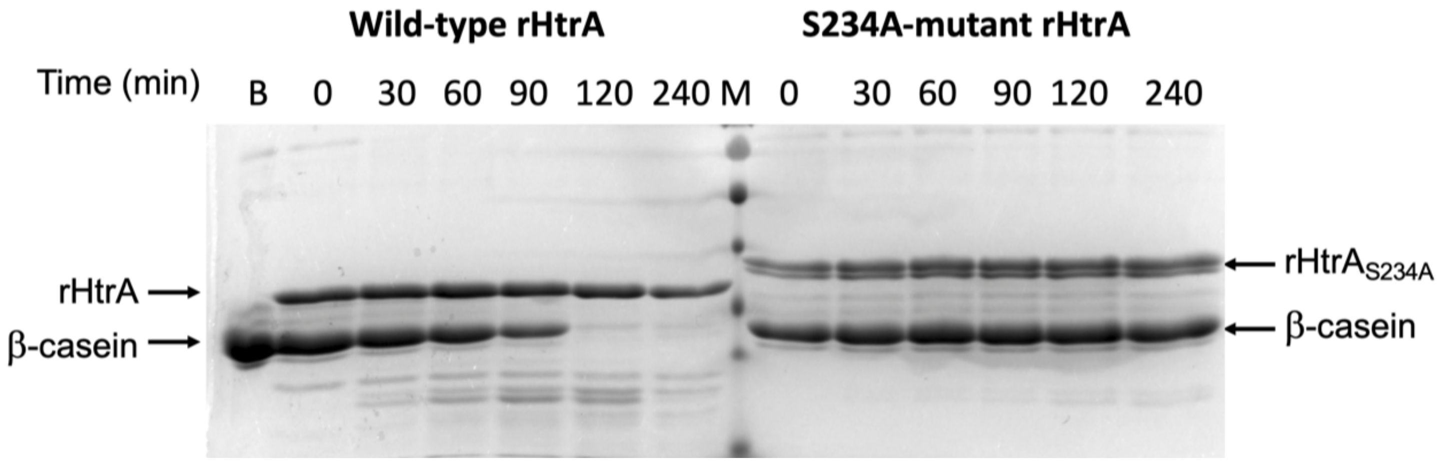

Supplementary Figure 4

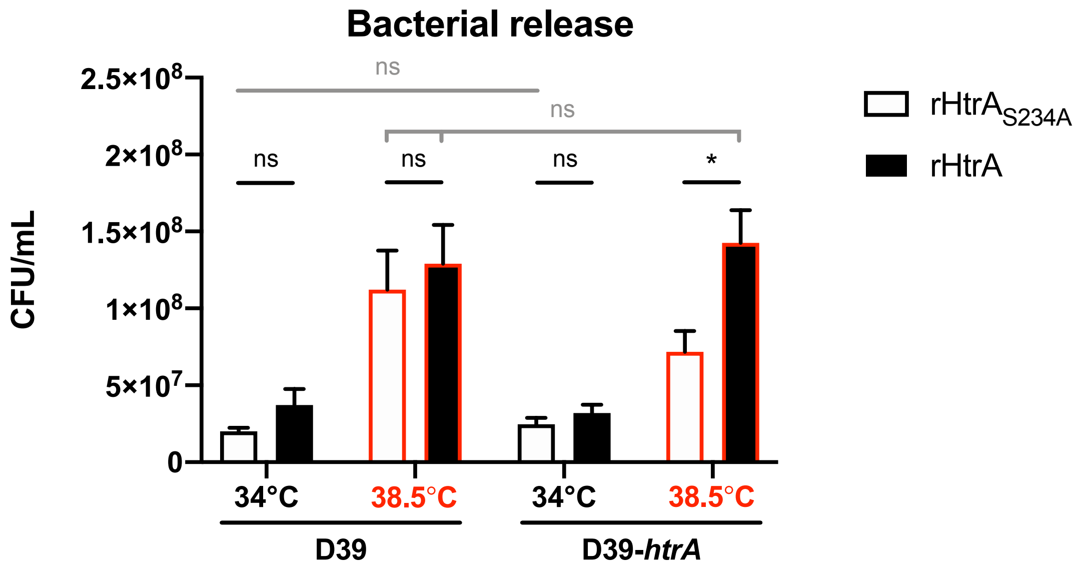

SUPPLEMENTARY REFERENCES

1 Ibrahim, Y. M., Kerr, A. R., McCluskey, J. & Mitchell, T. J. Role of HtrA in the virulence and competence of *Streptococcus pneumoniae*. *Infect Immun* **72**, 3584-3591, doi:10.1128/IAI.72.6.3584-3591.2004 (2004).

2 Sung, C. K., Li, H., Claverys, J. P. & Morrison, D. A. An rpsL cassette, janus, for gene replacement through negative selection in *Streptococcus pneumoniae*. *Appl Environ Microbiol* **67**, 5190-5196, doi:10.1128/AEM.67.11.5190-5196.2001 (2001).

3 Marks, L. R., Davidson, B. A., Knight, P. R. & Hakansson, A. P. Interkingdom signaling induces *Streptococcus pneumoniae* biofilm dispersion and transition from asymptomatic colonization to disease. *MBio* **4**, doi:10.1128/mBio.00438-13 (2013).

4 Li-Korotky, H. S., Lo, C. Y., Zeng, F. R., Lo, D. & Banks, J. M. Interaction of phase variation, host and pressure/gas composition: pneumococcal gene expression of PsaA, SpxB, Ply and LytA in simulated middle ear environments. *Int J Pediatr Otorhinolaryngol* **73**, 1417-1422, doi:10.1016/j.ijporl.2009.07.007 (2009).
